# Human Genetic Host Factors and Its Role in the Pathogenesis of Chikungunya Virus Infection

**DOI:** 10.3389/fmed.2022.654395

**Published:** 2022-02-16

**Authors:** Juan C. Rueda, Mauricio Arcos-Burgos, Ana M. Santos, Daniel Martin-Arsanios, Catalina Villota-Erazo, Viviana Reyes, Santiago Bernal-Macías, Ingris Peláez-Ballestas, Mario H. Cardiel, John Londono

**Affiliations:** ^1^Faculty of Medicine and Engineering, Universidad de La Sabana, Chía, Colombia; ^2^Grupo de Espondiloartropatías, Rheumatology Department, Universidad de La Sabana, Chía, Colombia; ^3^Grupo de Investigación en Psiquiatría (GIPSI), Departamento de Psiquiatría, Faculty of Medicine, Instituto de Investigaciones Médicas, Universidad de Antioquia, Medellín, Colombia; ^4^Rheumatology Department, Hospital Militar Central, Bogotá, Colombia; ^5^Rheumatology Unit, Hospital General de México “Doctor Eduardo Liceaga”, Mexico City, Mexico; ^6^Centro de Investigación Clínica de Morelia, Morelia, SC, Mexico

**Keywords:** chikungunya, genetic, host, pathogenesis, arbovirus

## Abstract

Chikungunya virus (CHIKV) is an alphavirus from the *Togaviridae* family that causes acute arthropathy in humans. It is an arthropod-borne virus transmitted initially by the *Aedes (Ae) aegypti* and after 2006's epidemic in La Reunion by *Ae albopictus* due to an adaptive mutation of alanine for valine in the position 226 of the E1 glycoprotein genome (A226V). The first isolated cases of CHIKV were reported in Tanzania, however since its arrival to the Western Hemisphere in 2013, the infection became a pandemic. After a mosquito bite from an infected viremic patient the virus replicates eliciting viremia, fever, rash, myalgia, arthralgia, and arthritis. After the acute phase, CHIKV infection can progress to a chronic stage where rheumatic symptoms can last for several months to years. Although there is a great number of studies on the pathogenesis of CHIKV infection not only in humans but also in animal models, there still gaps in the proper understanding of the disease. To this date, it is unknown why a percentage of patients do not develop clinical symptoms despite having been exposed to the virus and developing an adaptive immune response. Also, controversy stills exist on the pathogenesis of chronic joint symptoms. It is known that host immune response to an infectious disease is reflected on patient's symptoms. At the same time, it is now well-established that host genetic variation is an important component of the varied onset, severity, and outcome of infectious disease. It is essential to understand the interaction between the aetiological agent and the host to know the chronic sequelae of the disease. The present review summarizes the current findings on human host genetics and its relationship with immune response in CHIKV infection.

## Introduction

Chikungunya virus (CHIKV) is an alphavirus from the *Togaviridae* family, and a member of the Semliki Forest virus antigenic complex, that together with other alphaviruses (O'nyong-nyong, Mayaro, and Ross River) causes acute arthropathy in humans (1–3). The virion has an icosahedral capsid, enclosed by a lipid envelope with a single-stranded, positive sense, RNA genome of ~12 kilobases in length, which is arranged in two open reading frames (ORF) with a junction region in between (4, 5). The 5' ORF contains code for four non-structural proteins (nsP1-4), whereas the 3' encodes the capsid protein (C), two surface envelope glycoproteins (E1 and E2), and two small peptides designated E3 and 6k (6, 7).

A mosquito bite from an infected viremic patient is where the transmission initially starts (8). The virus replicates for a few days, before being transmitted to another person (9). Upon mosquito bite, the virus due to cellular tropism, infects fibroblasts in the dermis and macrophages, following an incubation period of 3–7 days, from where it is disseminated through lymphatics and bloodstream to joint capsule, muscle, epithelial, and endothelial cells (8). The virus replicates eliciting viremia, fever, rash, myalgia, arthralgia and arthritis (10). At this point the acute phase is established, lasting for ~2 weeks, and characterized by the appearance of immunoglobulin type M (IgM) which usually persist up to 3 months, however long-term follow up studies have demonstrated its presence after 10 months post infection in 17 to 76% of the patients and even up to 12 months (11–13). The production of immunoglobulin type G (IgG) starts usually 7–10 days post-onset of symptoms, which will provide antiviral immunity for years (5, 8, 10, 14). After the acute phase, CHIKV infection can progress to a chronic stage where rheumatic symptoms can last for several months to years (10).

CHIKV is an arthropod-borne virus transmitted initially by the *Aedes (Ae) aegypti* and after 2006's epidemic in La Reunion by *Ae albopictus* due to an adaptive mutation of alanine for valine in the position 226 of the E1 glycoprotein genome (A226V) (5, 15). It is believed that the infection started in a sylvatic or enzootic cycle where the virus was maintained through arboreal vectors (*Ae africanus*, and *Ae furcifer*) using non-human primates (vervet monkeys) as hosts, and later spilled over to humans living nearby forested regions (16). From here on, transmission from human to human via *Ae aegypti* or *albopictus* is amplified in urban settings and spread by air travel establishing an epidemic cycle (16).

The first isolated cases of CHIKV were reported in July 1952 along the coastal plateaus of Mawia, Makonde and Rondo, what is known today as Tanzania (17). The people of this region named the disease chikungunya, which translates to “the one that bends up the joints” (18). Phylogenetic studies indicated that CHIKV originated in Africa over 500 years ago and determined that a common lineage diverged into two branches termed West African (WA) and East/Central/South African (ECSA) (1, 2, 19, 20). While the ECSA lineage spread outside Africa causing multiple urban epidemics in Asia almost 150 years ago, WA lineage maintained local outbreaks in Africa through enzootic transmission (8, 20). It was in Asia were the ECSA lineage kept circulating and evolving into a separate genotype called Asian lineage (20). In the earlies 2000, the ECSA lineage reached Kenya and from there expanded to islands in the Indian Ocean, India, and Southeast Asia creating an unprecedented epidemic and again evolving into a new lineage with the aforementioned A226V mutation (Indian Ocean Lineage or IOL) (15, 21–23). This allowed the virus to use *Ae albopictus* as a new vector which has a higher altitude tolerance and therefore increasing the disease's reach to more temperate regions like southern France and northern Italy (24–27). During the last decade CHIKV continued to cause epidemics in the Pacific Islands, Indian subcontinent, Oceania and Southeast Asia (28–30). Finally, in 2013 the CHIKV Asian lineage arrived to the Western Hemisphere with the first autochthonous cases reported in the Island of Saint Martin (31). From there, the virus rapidly spread throughout the Caribbean, Central and South America, affecting 42 countries by 2015 (32). The virus kept circulating mainly in Asia and the Americas with outbreaks being continuously reported. In Brazil for example, 712.990 cases were accumulated over a period of 4 years since 2015 (33). In the years 2016, 2017 and 2019, a maximum number of laboratory confirmed cases were reported in India (34). Also, the first large epidemic in Pakistan was reported in December of 2016 with 1018 cases reported from multiple regions of Karachi (35). Up to September 2021, according to the European Center for Disease Prevention and Control, in 2021 133.928 cases have been reported (36). Of those, 106.768 from the Americas and the Caribbean (97% from Brazil), 27.056 from Asia (93% from India) and 104 from Africa (all from the Democratic Republic of Congo) (36).

Multiple studies have discovered genetic variants in the immune response to specific pathogens, providing important insights into the genetic control of immune signaling in humans (37). Increasing evidence support the fact that host genetics drives phenotypes of infectious diseases, however a gap still exists in CHIKV infection. A characteristic feature of many human infections is that only a proportion of exposed individuals develop clinical disease (37). Mouse studies have illustrated the potential importance of host genetic effects, by showing differences between different inbred strains in bacterial loads, cytokine responses and outcomes following bacterial and mycobacterial infection (38).

In humans, genetic studies, mainly using genome-wide association studies (GWAS), have discovered genetic variants in the immune response to specific pathogens, highlighting the role of shared host signaling pathways in the pathogenesis of diverse infectious diseases and providing important insights into the genetic control of immune signaling in humans (37). These studies have found correlations between gene polymorphisms with specific phenotypes in infectious diseases. For example, in HIV and AIDS, single nucleotide polymorphisms (SNP) in HLA-C, HLA-B and HCP5 genes were associated with high viral load at set point (39, 40). Also, dengue shock syndrome was associated in Vietnamese patients with SNPs in MICB and PLCE1 genes (41). Regarding CHIKV, few studies have been conducted to elucidate host genetics and specific phenotypes of the disease. The study of the genetic effects of CHIKV infected patients will increase our knowledge and understanding of the pathogenesis of the disease as well as the bases for potential treatment targets. The present review summarizes the current findings on human host genetics and its relationship with immune response in CHIKV infection.

## Pathogenesis

### Innate Immunity

Acute CHIKV infection elicits robust innate immune responses, leading to elevation of type I IFNs and numerous proinflammatory chemokines, cytokines, and growth factors (42–44). Type I IFN signaling controls viral replication and pathogenesis during acute infection (45, 46). In humans, IFN-α appears early in infection and correlates with viral load (42, 47). Coincident with rising viral loads and IFN-α responses, the vast majority of infected patients experience sudden onset of clinical illness, with a small proportion of infected individuals (5–28%) remaining asymptomatic (48).

**Figure 1:**
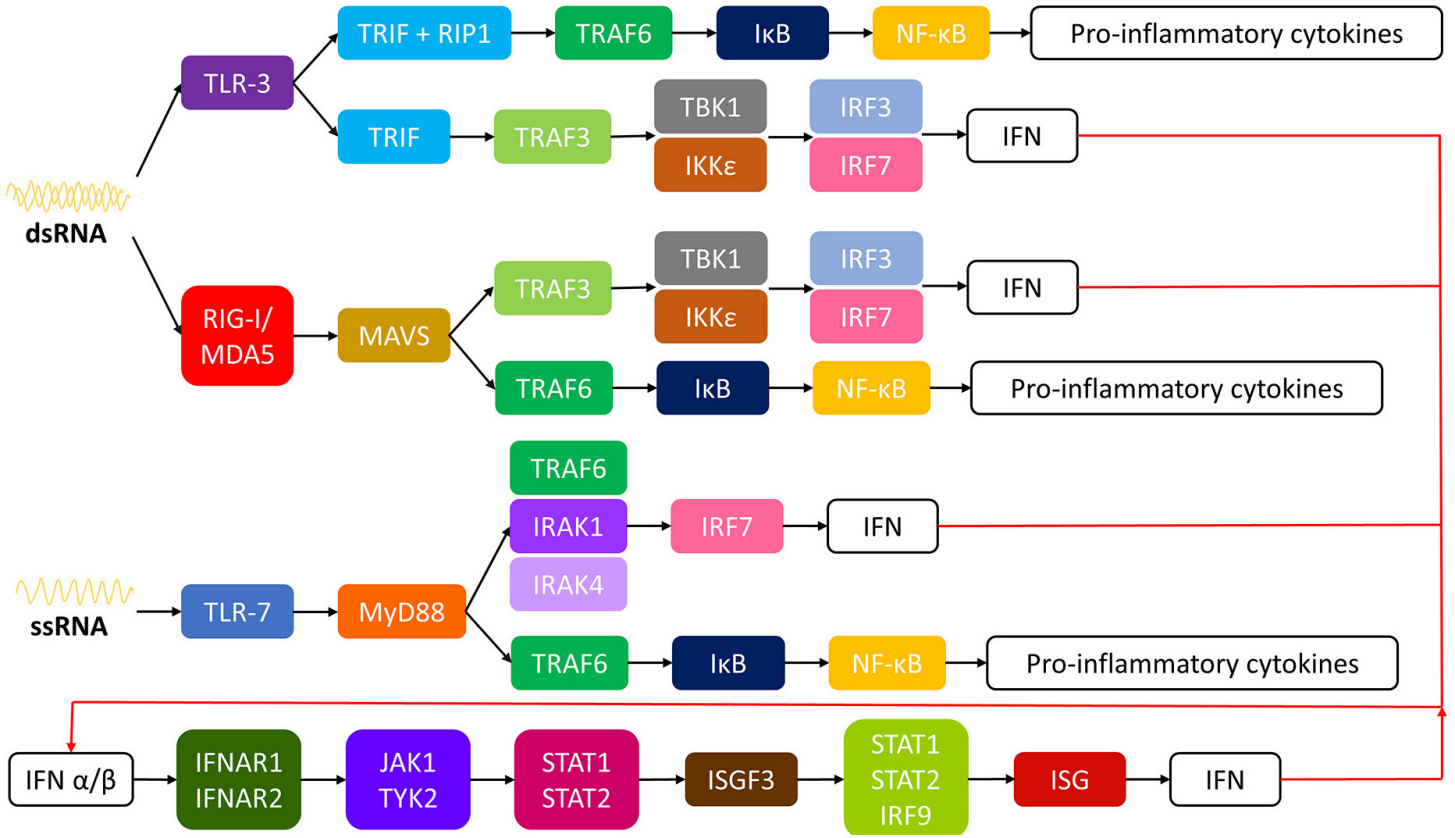
Intracellular pathways of innate immune response to CHIKV. CHIKV, chikungunya virus; ssRNA, single-stranded RNA; dsRNA, double-stranded RNA; TLR, toll like receptor; TRIF, TIR-domain containing adapter-inducing interferon-β; RIP-1, receptor interacting protein 1; TRAF, TNF receptor associated factor; IκB, inhibitor of κB; NF-κB, nuclear factor kappa-light-chain-enhancer of activated B cells; TBK1, tank binding kinase; IRF, interferon regulator factor; IFN, interferon; IKKε, inducible IκB kinase; RIG-I, retinoic acid-inducible gene I; MDAS, melanoma differentiation-associated protein 5; MAVS, mitochondrial antivirial signaling; MyD88, myeloid differentiation primary response gene 88; IRAK, interleukin-1 receptor-associated kinase; IFNAR, interferon α/β receptor; JAK1, Janus kinase 1; TYR2, tyrosine kinase 2; STAT, signal transducer and activator of transcription; ISGF3, ISG factor 3; ISG, interferon stimulated gene.

The mechanism which such immune response is initiated by CHIKV is like other emerging RNA virus infections. In a nutshell, innate immune response starts with recognition of viral RNA by pattern recognition receptors (PRRs). In the case of CHIKV, double stranded RNA (dsRNA) induce toll like receptor (TLR) 3, retinoic acid-inducible gene I (RIG-I) and melanoma differentiation-associated protein 5 (MDA5), while single-stranded RNA (ssRNA) induce TLR-7 (49–52). A signaling cascade is initiated which results in activation of nuclear factor kappa-light-chain-enhancer of activated B cells (NF-κB) and interferon regulator factors (IRFs) inducing the transcription of interferons and pro-inflammatory cytokines (49, 52–54). See Figure 1 for more detail. The produced type I interferons (IFN α/β) bind to receptors IFNAR1 and 2 (interferon receptor α/β) inducing phosphorylation of Janus kinase 1 (JAK1) and tyrosine kinase 2 (TYK2) which leads to assembly of interferon stimulated gene factor 3 (ISGF3) complex. ISGF3 binds to interferon stimulated genes (ISGs) resulting in gene expression of IFNs (52, 53, 55, 56). As expected, CHIKV developed mechanisms to counteract the host immune response. CHIKV inhibits IFN signaling with its own nsP2 by blocking the JAK/STAT (signal transducer and activator of transcription) signaling pathway (53, 57). Also, CHIKV nsP2 inhibits RNA polymerase II by inducing the degradation of its catalytic subunit RpB1, blocking the expression of cellular genes (53, 58, 59).

### Adaptive Immunity

Symptoms of acute CHIKV disease are caused by direct cellular damage and local inflammation, but the specific contributions of viral replication and the host immune response to CHIKV infection are yet to be completely unraveled (60). CHIKV infection is cytopathic and induces apoptosis, resulting in direct tissue injury. Numerous cell types, many of which are located at sites of disease, are susceptible to CHIKV, including chondrocytes, endothelial cells, fibroblasts, hepatocytes, macrophages, monocytes, muscle satellite cells, myocytes, and osteoblasts (61–64).

Monocytes and macrophages are targeted by CHIKV and contribute to virus-induced pathogenesis. Activated macrophages are the primary infiltrating cell in infected tissues (65, 66), and elevated levels of MCP-1, the major chemoattractant for monocytes and macrophages, correlate with high viral loads in persons with acute CHIKV infection (42–44, 47, 66, 67). A meta-analysis of immune mediators from geographically distinct cohorts revealed an immune mediator signature dominated by proinflammatory cytokines, which include INF-α, INF-γ and Il-2, 2R, 6, 7, 12, 15, 17 and 18 (42).

Acute infection in humans leads to activation and proliferation of CD8+ T cells, while a CD4+T cell response is dominant during the chronic phase of CHIKV disease (68). Although activated, CD8+ T cells do not appear to mediate CHIKV clearance or disease in animals (69). In contrast, studies using mice deficient in various types of lymphocytes implicate CD4+ T cells as inflammatory mediators in infected tissues (70). However, these cells also may contribute to viral clearance (70). Tregs are involved in CHIKV pathology, as expansion of Tregs reduces CHIKV disease by selectively inhibiting CHIKV-specific CD4+ effector T cells (71). In addition, γδ T cells, which are abundant in skin, protect against CHIKV disease, as γδ T cell–deficient mice display exacerbated CHIKV infection (72). Development of CHIKV neutralizing antibodies is essential to control CHIKV viremia (69, 70). In humans, IgM levels are detected within 5–7 days after the onset of symptoms, peak several weeks after infection, and begin to wane over the next several months (14). An IgG response can be detected approximately 7–10 days after onset of illness, often after viremia has been cleared (14).

Studies of CHIKV-infected humans and animals have defined symptoms and immune responses of acute CHIVK disease, but much of the molecular interplay between virus and host remains to be established. To this date, it is unknown why a percentage of patients do not develop clinical symptoms despite having been exposed to the virus and developing an adaptive immune response (presence of positive CHIKV IgM or IgG).

## Host Genetics and Chikungunya

### Human Leukocyte Antigen

Virulence factors specific to the infective agent as well as host factors like innate and adaptive immune response play an important part in disease susceptibility (73–75). Regarding host factors, the HLA plays a major role in initiating immune responses; the HLA type I molecules present pathogen peptides to CD8+ lymphocyte cells, while HLA type II molecules present pathogen peptides to CD4+ lymphocyte cells (76).

HLA molecules are coded on the short arm of chromosome 6, occupying a large portion of the DNA (~3,500 kilobases) (77). To date, HLA molecules are the most polymorphic genes in the human genome (77). These polymorphisms allow the immune system to increase the repertoire of peptides presented by HLA molecules, which in turn will affect the susceptibility to infectious diseases.

**Figure 2:**
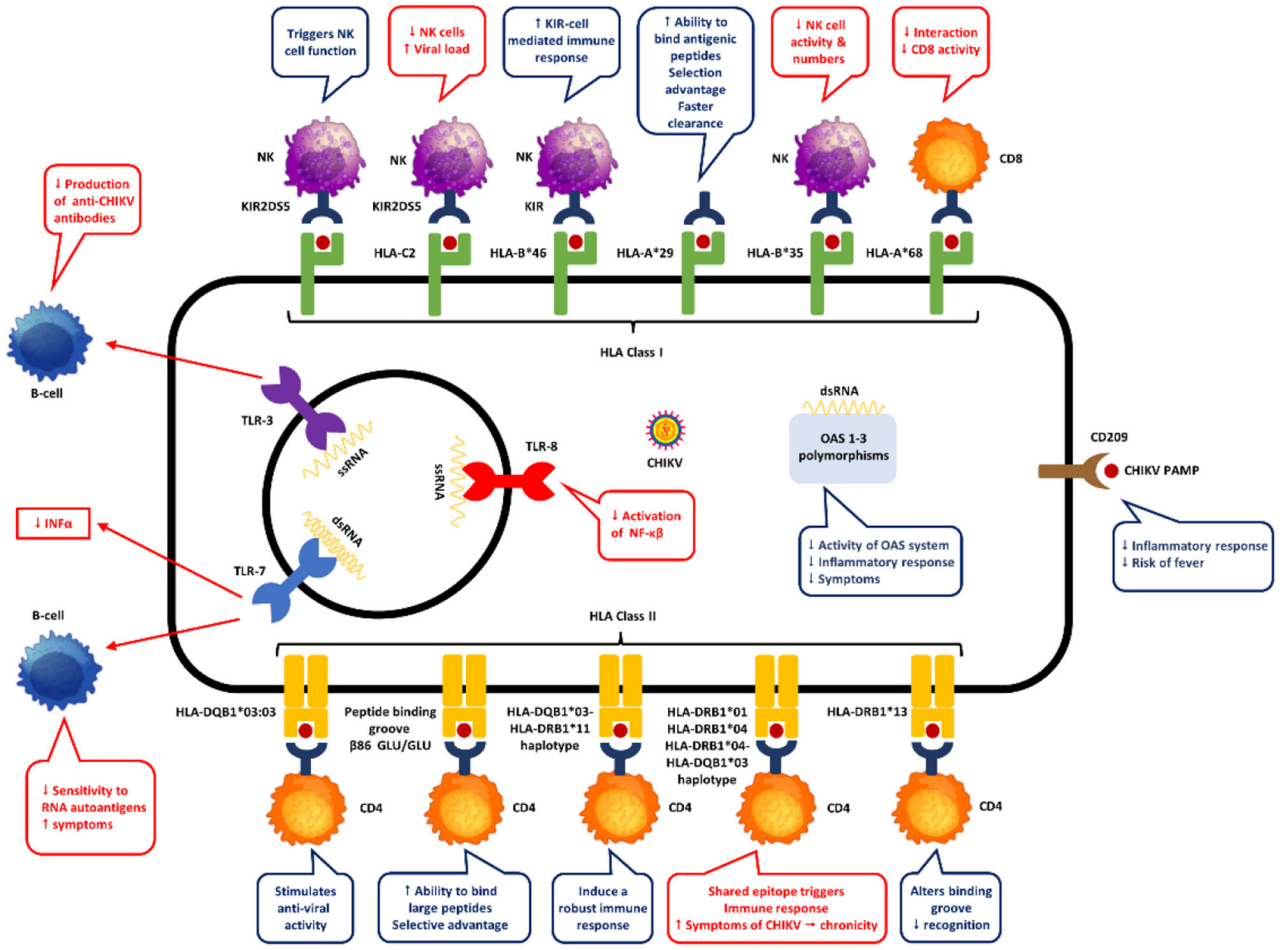
Proposed mechanisms of genetic host factors in CHIKV pathogenesis.

Many chronic diseases and their host's interaction with the disease have been studied to understand their immunogenetics. HLA-DR3 (HLA- DRB1^*^0301, DQB1^*^0201) and DR4 (HLA-DRB1^*^04, DQB1^*^0302) are markers for type 1 diabetes in Caucasian populations, but not among Japanese patients (78). The DRB1^*^07 allele is associated with protection against Dengue virus infection in the Cuban population, but not in the Sri Lankan population (79, 80). Studies have demonstrated HLA class II alleles association to susceptibility or resistance to CHIKV (Table 1, Figure 2), however none have reported associations with clinical symptoms of CHIKV infection (78, 81, 82).

**Table 1:** Genetic studies on host and CHIKV infection.

**Country**	**Sample**	**Gene**	**Allele/polymorphism**	**Findings**			**Type of association**	**Year**	**Reference**
				**OR**	**CI**	***p***			
India	- 101 CHIKV - 104 controls	HLA class II - DQB1 - DRB1	- DQB1*03:03 - Peptide binding groove DQ *β86 non GLU *β86 GLU/GLU	0.13 1.79 0.30	0.04-0.40 1.16-2.74 0.12-0.70	0.024 0.008 0.004	Protection against CHIKV Susceptibility to CHIKV Protection against CHIKV	2013	(78)
India	- 100 CHIKV - 250 controls	HLA class II - DQB1 - DRB1	- DRB1*11 - DRB1*11/DQB1*03 - DRB1*04/DQB1*03	0.21 0.15 1.94	0.07-0.61 0.03-0.66 1.06-3.55	0.002 0.007 0.042	Protection against CHIKV Protection against CHIKV Susceptibility to CHIKV	2014	(81)
Gabon	- 73 CHIKV - 54 controls	KIR HLA class I - C - Bw4	- 2DL1 - 2DS5 - C2-2DL1 - Bw4-80Trn-3DL1	NP NP NP NP	NP NP NP NP	0.033 0.050 0.023 0.001	Susceptibility to CHIKV Protection against CHIKV Susceptibility to CHIKV Protection against CHIKV	2014	(83)
Singapore	- 94 CHIKV - 179 controls	TLR-3	- RS3775292 - RS6552950 - RS6552950	2.16 1.54 2.31	1.31-3.42 1.03-2.29 1.16-4.57	0.002 0.03 0.02	Susceptibility to CHIKV Susceptibility to CHIKV Susceptibility to severe disease	2015	(84)
India	- 101 CHIKV - 101 controls	CD209 OAS1 OAS2 OAS3	- CD209 *RS4804803 - A-G - OAS1 *RS1131454 - G-G - OAS2 *RS1732778 - A-A *RS15895 - G-A *RS15895 - G-A - OAS3 *RS2285932 - C-T *RS2285832 - T	0.16 0.32 0.16 0.34 0.39 0.39 0.40	0.03-0.97 0.11-0.96 0.05-0.57 0.13-0.88 0.15-0.99 0.16-0.92 0.17-0.91	0.037 0.036 0.002 0.024 0.036 0.031 0.028	Protection to fever Protection to oedema Protection to nausea Protection to chills Protection to red eye Protection to chills Protection to oedema	2016	(85)
India	- 173 CHIKV - 157 controls	TLR-3 TLR-7 TLR-8	- TLR-3 *RS3775290 - T-T - T-T - C - C-T - TLR-7 *RS179008 - C-C - T-C *RS5741880 - G-T - G-T - G-G *RS3853839 - G-C - G-C - G-C - G-C - C-C - C - C *RS179010 - C-C - C-C - C-C - T-C - T-C - T-T - TLR-8 *RS53764879 - G-C - G-C - G-C - G - C-C *RS3764880 - G-G	2.10 0.30 0.53 5.05 2.03 0.43 0.43 0.33 6.84 4.33 3.44 2.50 0.42 10.0 2.82 2.28 2.03 2.42 3.77 0.43 0.33 0.36 3.04 2.63 3.31 0.68 0.19 2.04	1.03-4.30 0.14-0.76 0.34-0.84 1.55-11.1 1.12-3.66 0.25-0.74 0.23-0.76 0.13-0.86 0.90-51.4 2.23-8.53 2.12-5.57 1.23-5.07 0.22-0.78 0.51-196 1.84-4.32 1.19-4.35 1.12-.3.66 1.08-5.41 1.39-10.1 0.25-0.74 0.15-0.76 0.17-0.76 1.83-5.05 1.31-5.29 1.60-6.84 0.47-0.97 0.04-0.91 1.06-3.90	0.039 0.002 0.007 0.003 0.017 0.002 0.030 0.019 0.032 0.000 0.006 0.009 0.050 0.039 0.000 0.010 0.010 0.028 0.003 0.002 0.002 0.002 0.000 0.005 0.000 0.034 0.020 0.031	Susceptibility to CHIKV^†^ Protection to fever Protection against CHIKV^†^ Susceptibility to joint pain Susceptibility to CHIKV Protection against CHIKV Protection against CHIKV Protection against CHIKV^†^ Susceptibility to joint pain Susceptibility to CHIKV^†^ Susceptibility to CHIKV Susceptibility to CHIKV^‡^ Protection to rash Susceptibility to CHIKV Susceptibility to CHIKV Susceptibility to CHIKV^‡^ Susceptibility to CHIKV Susceptibility to CHIKV^‡^ Susceptibility to high INFα Protection against CHIKV Protection against CHIKV^‡^ Protection against fever Susceptibility to CHIKV Susceptibility to CHIKV^‡^ Susceptibility to CHIKV^†^ Protection against CHIKV Protection against CHIKV^†^ Susceptibility to fever	2017	(86)
Colombia	- 65 CHIKV - 100 controls	HLA-class I - A - B HLA-class II - DRB1	- A*29 - A*68 - B*35 - B*46 - DRB1*01 - DRB1*04 - DRB1*13	0.10 8.90 2.02 0.26 5.70 7.37 3.75	0.02-0.44 1.8-42.1 1.06-3.86 0.10-0.67 1.9-16.5 3.3-16.3 1.50-9.39	0.002 0.005 0.030 0.005 0.001 0.000 0.004	Protection against CHIKV Susceptibility to CHIKV Susceptibility to CHIKV Protection against CHIKV Susceptibility to CHIKV Susceptibility to CHIKV Susceptibility to CHIKV	2021	Accepted

*OR, odds ratio; CI, 95% confidence interval; CHIKV, chikungunya virus infection; HLA, human leukocyte antigen; KIR, killer cell immunoglobulin like receptor; TLR, toll like receptor; OAS, 2'-5'oligoadenylate synthase*;

† *: in females*;

‡ *: in males*.

A study in 73 Gabonese patients with CHIKV infection, Petitdemange et al. studied the association between killer cell immunoglobulin like receptors (KIR), their HLA-class I genotype and susceptibility to CHIKV (83). KIR receptors on the surface of natural killer (NK) cells bind to HLA-class I molecules on target cells. The KIR-HLA interaction has a functional significance in response to infectious diseases activating or inhibiting NK cells. The authors found that KIR2DL1 and its interaction with HLA-C2 (KIR2DL1-C2) granted susceptibility to CHIKV infection, while KIR2DS5 conferred protection (83). The authors believe that the expansion of highly functional NK cells and the development of a strong adaptive memory response are associated with the interplay between KIR2DL1 and HLA-C2 in CHIKV infected patients.

In a study of 21 patients from Reunion Island with chronic chikungunya, HLA-DRB1^*^01 and DRB1^*^04 alleles were frequently found among the patients who developed rheumatoid arthritis after the infection, indicating the probable involvement of HLA class II gene in chikungunya infection (87). However, details regarding the relevance of these alleles with chikungunya virus associated clinical presentations were not indicated in the study.

In India, two studies have reported the association of HLA alleles with CHIKV infection (78, 81). One study demonstrated the presence of HLA-DRB1^*^04-HLA-DQB1^*^03 haplotype with susceptibility to CHIKV infection, and the presence of HLA-DRB1^*^11 and HLA-DRB1^*^11-HLA-DQB1^*^03 haplotype with resistance to the infection (81). According to the authors, one explanation could be that the epitopes presented by HLA-DRB^*^11 modulates toward a more robust immune response to CHIKV by means of CD4 T cells.

The other study reported a lower frequency of HLA-DQB1^*^03:03 allele in CHIKV infected patients with statistical significance when compared to normal subjects (78). The authors suggest that the DQ^*^03:03 allele may stimulate diverse antiviral CD4 T-cell responses and therefore its association with protection against CHIKV. In this study, although HLA-DRB1^*^01 and HLA-DRB1^*^04 were more frequent in CHIKV infected patients, the difference was not statistically significant. In the same study, the presence of non-glutamic acid at position 86 of the peptide biding groove of DQB1genotypes was associated with infection while lower frequencies of glutamic acid at the same position was protective against CHIKV infection when compared with healthy controls. This can be explained by the fact that the presence of glutamate at the β86th position allows for accommodation of peptides with large hydrophobic amino acids, which in turn enhance the ability to bind a large number of amino acids conferring a selective advantage against CHIKV (78, 88). Taking this into account, special interest should be given to research of HLA-DRB1 alleles in CHIKV infected patients and its role in the pathophysiology of the disease.

A study on 65 patients with confirmed CHIKV infection from Colombia demonstrated the presence of five HLA class I and II alleles in CHIKV infected patients when compared to healthy subjects (accepted). Specifically, HLA-A^*^68, HLA-B^*^35, HLA-DRB1^*^01, HLA-DRB1^*^04 and HLA-DRB1^*^13. Of interest, HLA-A^*^68 and HLA-DRB1^*^04 had the strongest association with CHIKV infection. In fact, HLA-DRB1^*^04 was the only allele associated with the presence of rash in the abdomen and the face. No other alleles were statistically associated with other clinical symptoms or disability scores. Only class I alleles (HLA-A^*^29 and HLA-B^*^46) were associated with reduced risk of CHIKV infection as well as rash in the abdomen and the face (HLA-B^*^35). None of the HLA-DRB1 alleles showed protection for CHIKV infection. Studies have found that in HLA-A^*^68 a valine residue at position 245 replace the alanine present in other HLA molecules (89, 90). This distorts the α3 loop, resulting in a less energetically favorable interaction with CD8-T cells, which could explain the susceptibility to infection in CHIKV patients. On the other hand, HLA-A^*^29 distinguishes from other HLA-A alleles by the presence of leucine at position 62 and glutamine at position 63 (91). This change in position 63 has the largest effect on the ability to bind antigenic peptides in the peptide binding groove of HLA-A (91). In fact, the 62–63 motif may influence the flexibility to accommodate antigenic peptides, which can confer selective advantage against CHIKV (91). In another study in human immunodeficiency virus (HIV) infected patients, the progression to AIDS was associated with the presence of HLA-B^*^35-HLA-Cw4 haplotype due to reduction of natural killer cell number and activity (92). A finding that could explain the susceptibility to CHIKV infection in our population. In our population, HLA-B^*^46 was associated with protection against CHIKV infection. HLA-B^*^46 is an unusual allele formed by the recombination between HLA-B^*^15 and HLA-C^*^01, which allows it to interact with KIR ligand (93). Its distinctive peptide-binding site derived from its recombination and ability to react with KIR ligands helps create a strong KIR cell mediated immune response, which in turn could explain its protection against CHIKV infection. Regarding HLA class II alleles, HLA-DRB1^*^01 and ^*^04, which were associated with susceptibility to CHIKV in our population, were also found in parvovirus B19 infection by Kerr et al. (94). It is known that both DRB1^*^01 and ^*^04 code for the shared epitope in residues 70 to 74 in the HLA-DRB chain (95). We hypothesize that CHIKV antigens could bind the shared epitope triggering innate immune signaling as well as production of pro-inflammatory cytokines like IL-6, TNF-α and IL-23 by dendritic cells, conferring symptomatic susceptibility to CHIKV infection. Finally, HLA-DRB1^*^13 has been associated with carrying an asparagine in position 37 of P9 peptide binding pocket granting electropositibity (96). This restricts the range of peptides that can be presented which is sufficient to alter recognition by the T-cell receptor, and therefore increase susceptibility to CHIKV infection.

The relationship between host genetics and disease can be variable. The presence of an allele like HLA-DRB1^*^13 is associated with CHIKV infection (susceptibility), on the other hand, the same allele confers protection for AIH. On the contrary, while HLA-A^*^29 and B^*^46 protect against CHIKV infection, they are associated with birdshot retinochoroidopathy and severe sacroiliitis in psoriatic arthritis, respectively. To explain the relationships between HLA variants involved in both autoimmune and infectious diseases, two hypotheses have been proposed (97). The first hypothesis states that pressure on the human genome by pathogens has led to selection of host defense genes that protects against infections. However, this advantageous selection may also increase the risk of developing autoimmune diseases. The second hypothesis suggests that pathogens can trigger autoimmunity by molecular mimicry, epitope spreading bystander activation or cryptic antigens (98).

### Candidate Genes

Genes outside the HLA complex have been studied in CHIKV infection (Table 1, Figure 2). Specifically, polymorphisms of TLR genes as well the 2'-5'-oligoadenylate synthetase (OAS) gene cluster and CD209. As mentioned before, tt is well-established that the innate immune response starts with recognition of viral RNA genome by PRRs. In the case of CHIKV infection, its RNA is recognized by TLRs (3, 7 and 8), retinoic acid-inducible gene I (RIG -I) like receptors (RLRs) and melanoma differentiation-associated protein 5 (MDA5) (99). The PRRs stimulates the production of pro-inflammatory cytokines through myeloid differentiation factor 88 (MyD88) and TRAF, which in turn activates OAS genes increasing viral degradation (62). TLR-3 polymorphisms can change the ecto-domain of the receptor altering the ligand-receptor interaction, while TLR-7 polymorphisms affects TLR-7 processing and receptor expression (100–102). Studies on TLR-8 polymorphisms have demonstrated variation in transcription of TLR-8 isoforms which affects NF-κB activation as well as diverse production of TNF-α and IL-1β (103, 104).

A study found the SNP rs6552950 in the TLR-3 gene to be associated with disease severity and CHIKV-specific neutralizing antibody response and the SNP rs3775292 to disease susceptibility (84). In this study the authors demonstrated that the polymorphisms in TLR-3 gene produced a loss in TLR-3 functionality which decreased the production of anti-E2EP3 IgG anti-bodies (CHIKV specific neutralizing antibodies), reducing CHIKV clearance and eliciting severe disease (defined by maximum temperature >38.5°C, a maximum pulse rate >100 beats/min or a nadir platelet count <100 x 10^9^/l).

Similarly, another study found three polymorphisms of TLR-7 (rs179010, rs5741880, rs3853839) and one of TLR8 (rs3764879) to be significantly associated with CHIKV infection (86). In the same study, the SNP rs3775290 from the TLR-3 gene was associated with the presence of joint pain, the SNPs rs179010, rs5741880 and rs38533839 from the TLR-7 gene with fever, joint pain, and rash, respectively. Interestingly, the SNP in the TLR-7 gene associated with fever (rs179010) was also present in patients with CHIKV infection and increased levels of IFN-α. Finally, fever was also associated with a SNP (rs3764880) in the TLR-8 gene. Studies have found that polymorphisms in TLR-7 increases B-cell sensitivity to RNA-containing autoantigens in the development of systemic autoimmunity. Specifically, rs3853839 of TLR-7 in lupus patients was associated with skin involvement like malar rash and photosensitivity (105). Also, TLR-7 and−8 polymorphisms may impair the immune response to hepatitis C virus due to less INF-α (103). Other studies have demonstrated that TLR-8 polymorphisms could produce a truncated TLR-8 with a shorter signal peptide which result in a more rapid decay of TLR-8 or may affect the protein function impairing NF-κB activation *in-vitro* (106, 107).

The OAS gene cluster and CD209 (also known as DC-SIGN: dendritic cell-specific intercellular adhesion molecule-3-grabbing non-integrin) has also been studied in CHIKV infected patients. OAS activation by INFs degrades viral RNA decreasing viral replication, while the DC-SIGN, a type II transmembrane in macrophages and dendritic cells plays an important role in innate immunity activation through recognition of high-mannose type N-glycans pathogen associated molecular patterns (PAMPs) (108–111). A study in India found that the rs4804803 of the CD209 gene is associated with susceptibility to CHIKV infection (85). The researchers also found that the same polymorphism (rs4804803) and others from de OAS gene cluster (OAS1 rs1131454, OAS2 rs1732778 and rs15895, and OAS3 rs2285932) influence the risk of developing clinical symptoms in CHIKV infected patients. The authors hypothesize that some OAS polymorphisms reduce the activity of the OAS system, decreasing inflammatory response which could protect against developing symptoms. On the other hand, other OAS polymorphisms increase antiviral activity, reducing viral replication and therefore protecting against developing symptoms. Also, the authors believe that CD209 polymorphisms affect binding of transcription factors and the presence of G-G polymorphism influence the expression of DC-SIGN, all of which at end affects the innate and adaptive immune response of dendritic cells increasing susceptibility to CHIKV infection. Conversely, G-A polymorphisms of CD209 decreases inflammatory response which could explain its association with protection against fever.

## Conclusion

Increasing evidence support the fact that host genetics drives phenotypes of infectious diseases, however a gap still exists in CHIKV infection. So far it has been shown that the presence of certain alleles in certain populations as well as polymorphisms in certain genes change the presentation and course of the disease. However, some important questions remain unanswered. Why some patients develop symptoms, while others do not regardless of being infected? Why some patients develop chronic symptoms? Why the host immune system responds differently to the same infectious agent? Is there a genetic predisposition to host-agent interaction? Questions that can be applied not only to CHIKV but to other infectious diseases. The door remains open for research to provide a newer insight in the host dynamics and infectious diseases, especially, by conducting association studies between inflammatory markers, clinical manifestations, with long-term follow-up are necessary to understand the repercussions that host genetic factors have on the immune response and how this is reflected in the clinical picture of the infection both acutely and chronically. Additionally, the possibility of conducting genetic studies that include not only candidate genes and HLA alleles using genome wide analysis or exome wide analysis techniques in conjunction with proteomic techniques will allow us to find possible associations that at first glance were not on the radar of the pathogenesis of the disease. The study of the genetic effects of CHIKV infected patients will increase our knowledge and understanding of the pathogenesis of the disease as well as the bases for potential treatment targets.

## Author Contributions

JR, MA-B, AS, DM-A, CV-E, VR, SB-M, IP-B, MC, and JL: conceptualization, supervision, funding acquisition, methodology, data curation, writing-original draft preparation, and writing-review and editing. All authors contributed to the article and approved the submitted version.

## Funding

The study was supported by the Colombian Rheumatology Association (ASOREUMA) under grant number Acta 169 10th July 2015; Universidad de La Sabana under grant number MED-197-2015; and COLCIENCIAS doctoral scholarship under grant number 757-2016.

## Conflict of Interest

The authors declare that the research was conducted in the absence of any commercial or financial relationships that could be construed as a potential conflict of interest.

## Publisher's Note

All claims expressed in this article are solely those of the authors and do not necessarily represent those of their affiliated organizations, or those of the publisher, the editors and the reviewers. Any product that may be evaluated in this article, or claim that may be made by its manufacturer, is not guaranteed or endorsed by the publisher.
